# Digital Intervention (MiVacunaLA 2.0) to Promote COVID-19 Vaccine Acceptance Among Hispanic Children: Community-Based Randomized Controlled Trial

**DOI:** 10.2196/78103

**Published:** 2026-03-30

**Authors:** Luisa R Blanco, Alexandra Klomhaus, Rebecca Dudovitz, Denise Marquez, Ray Lopez-Chang, Evelyn Aleman, Bas Weerman, Michael Moldoff, Keith Norris, Yelba Castellon-Lopez

**Affiliations:** 1 School of Public Policy, Pepperdine University, 24255 Pacific Coast Highway, Malibu, CA, 90263, United States, 1 310-506-7466; 2 Department of Medicine, David Geffen School of Medicine, Division of General Internal Medicine and Health Services Research, University of California, Los Angeles, Los Angeles, CA, United States; 3 Department of Pediatrics, David Geffen School of Medicine, Division of General Pediatrics, University of California, Los Angeles, Los Angeles, CA, United States; 4 Self-Sufficiency Services, Office of Immigrant and Refugee Affairs, County of San Diego, Health and Human Services Agency, San Diego, CA, United States; 5 Center for Economic and Social Research, USC Dornsife, University of Southern California, Los Angeles, CA, United States; 6 Our Voice: Communities for Quality Education (Nuestra Voz), Los Angeles, CA, United States; 7 Cancer Research Center for Health Equity, Samuel Oschin Comprehensive Cancer Institute, Department of Biomedical Sciences, Cedars-Sinai Medical Center, Los Angeles, CA, United States

**Keywords:** children digital education, Latino/Hispanic children, health equity, COVID-19 vaccines, mobile-based interventions

## Abstract

**Background:**

Early in the children’s COVID-19 rollout in the United States, racial and ethnic vaccination rate disparities were evident. Based on COVID-19 communication literature and qualitative interviews with Hispanic parents, we developed a mobile phone–delivered digital intervention to address factors associated with low vaccine confidence.

**Objective:**

We conducted a community-based randomized controlled trial of a digital intervention called MiVacunaLA/MyShotLA to increase COVID-19 vaccine uptake among Hispanic children. The fully automated digital intervention was designed in collaboration with community organizations and linguistically and culturally tailored to meet the informational needs of Hispanic caregivers. The intervention focused on families with unvaccinated children 5 to 11 years old but was offered to families with any unvaccinated children 17 years or younger.

**Methods:**

Participants were recruited with community organization partners and trained parent ambassadors via an open online screener. The 4-week intervention consisted of 3 SMS text messages with culturally and linguistically tailored educational information weekly. Intervention materials were delivered digitally through a closed online platform. Study team members were blinded. We used a difference-in-difference model with an intention-to-treat approach. The primary outcome was self-reported COVID-19 vaccine uptake among household children collected via online questionnaires. Secondary outcomes included COVID-19 vaccine knowledge, vaccine trust, and measures of participant engagement. We conducted a sensitivity analysis using the treatment-on-the-treated approach.

**Results:**

In total, 254 participants completed the baseline survey (128 control and 126 intervention). The average participant age was 34 (SD 6.3) years with an average of 1.7 (SD 0.8) minors in the household, and among households, 62.2% (n=158) reported having children aged 5 to 11 years old. Most participants (n=207, 81.5%) reported English as their primary language. We found a statistically significant difference of 13.3% (95% CI 0.3%-26.4%; *P*=.04) points in self-reported vaccine uptake between intervention and control groups among caregivers of Hispanic children aged 5 to 11 years old. We also found a statistically significant point difference of 14.3% (95% CI 0%‐23.7%; *P*=.003) between intervention and control groups in trust of governmental approval processes for the children’s COVID-19 vaccine. Most participants reported that the weekly digital videos and educational information were “very” (892/1031, 86.5%) or “extremely” (888/1019, 87.1%) useful.

**Conclusions:**

MiVacunaLA demonstrates that a culturally tailored, community-based, mobile phone–delivered vaccine educational intervention can increase COVID-19 vaccine uptake among Hispanic children and improve caregivers’ trust in governmental vaccine processes. MiVacunaLA is innovative in its integration of community-informed design with a fully automated, mobile phone–centric format and builds on prior literature by prospectively evaluating a culturally tailored, SMS text messaging–linked web curriculum in a community setting. Findings provide evidence that scalable, low-cost, digital strategies can measurably improve trust and uptake in a population facing persistent vaccination gaps. Real-world implications include the portability and adaptability of this approach across diverse communities and settings to support timely, community-engaged vaccination efforts for broader applicability and scalability in public health.

## Introduction

### Background

During the initial rollout of the COVID-19 vaccine for children of ages 5 to 11 years in the United States, children identifying as part of a racial and ethnic minority group were underrepresented among first-dose recipients [1-5]. This was due, in part, to lack of access to accurate information, concerns about vaccine safety and efficacy, exposure to disinformation and misinformation, and lack of trust in governmental vaccine processes [5-8]. Studies show that parental and caregiver acceptance of COVID-19 vaccination for their children is positively associated with beliefs about vaccination benefits [9]. Improving parental and caregiver acceptance is a key public health focus, given the trends in childhood vaccine uptake after the COVID-19 pandemic.

Following the pandemic, vaccination rates among US kindergartners across other childhood vaccinations declined compared to prior years [10,11]. Meanwhile, the United States has seen the resurgence of previously eliminated or highly controlled vaccine-preventable diseases, such as measles-mumps-rubella (MMR) [12,13]. While prepandemic vaccination rate disparities existed among racial and ethnic subgroups [14], these trends were further exacerbated by the pandemic [15]. As such, novel interventions are needed to effectively combat disinformation and misinformation, rebuild trust in vaccination benefits and safety, and engage undervaccinated communities.

In the early stages of the COVID-19 vaccine rollout, leveraging community-engaged strategies aided in promoting vaccine confidence and addressing barriers such as language accessibility, addressing disinformation and misinformation, and building trust in vaccination among the Hispanic population [16,17]. Incorporating community voices and perspectives into vaccine promotion and distribution strategies can have more meaningful impact with community members as opposed to traditional communication methods [18,19]. Digital interventions have also increased uptake of other vaccines, such as human papillomavirus vaccination, suggesting that community-based digital interventions might be particularly effective at increasing vaccine confidence and uptake to address recent declines in children vaccination rates [20,21]. However, few randomized controlled trial (RCT) studies exist on the use of community-based digital interventions to improve COVID-19 vaccination rates among Hispanic children. Testing and identifying such interventions can yield critical public health and primary care approaches and lessons for responding to both current vaccine uptake disparities and future public health emergencies.

### Study Objectives

This study describes efforts to increase COVID-19 vaccination rates among Hispanic children of ages 5 to 11 years in the Los Angeles County area through a community-informed culturally and linguistically appropriate RCT. We evaluated the efficacy of a digital intervention, called MiVacunaLA/MyShotLA (MVLA) 2.0, for parents or caregivers of Hispanic children to increase COVID-19 vaccination uptake for children. We also evaluated the intervention’s efficacy in increasing knowledge of COVID-19 vaccines as well as trust in the governmental approval process for the vaccine.

## Methods

### Intervention Content and Approach

MVLA 2.0 builds on MVLA 1.0, which was created in response to the US Food and Drug Administration–approved COVID-19 vaccines for children of ages 12 to 17 years and COVID-19 communication literature [22]. The MVLA 1.0 design and implementation are described in detail elsewhere [22]. Briefly, MVLA 1.0 used a community-partnered approach to develop and distribute COVID-19 vaccine education via SMS text messaging, emails, and brief videos multiple times a week for 4 weeks. MVLA 2.0, the focus of this study, began recruitment between June and August of 2022, with the intervention implemented between August and November 2022.

For MVLA 2.0, we updated and refined the digital materials to focus on vaccination for children aged 5 to 11 years based on parental and community partner feedback. We conducted focus groups with parents and caregivers of children between the ages of 5 and 11 years from MVLA 1.0 to tailor MVLA 2.0 for the 5- to 11-year-old age group. Detailed focus group results are reported in a separate paper [23] and revealed the need to adapt the intervention’s educational content to address emerging COVID-19 vaccine concerns specific to children, as well as myths and misinformation [23]. Next, we collaborated with community-based organizations to conduct community sessions with parent advocates from these organizations. Participants suggested several new features to MVLA that included (1) infographics to visually summarize weekly content, (2) voice-overs in English and Spanish of text to address literacy needs, (3) a discussion board where participants could post questions about the vaccine and have a physician respond, and (4) parent testimonials from those who had vaccinated their children. In addition, community partners identified the need for new content to address questions about emerging COVID strains. These suggestions were all incorporated into the MVLA 2.0 intervention.

For the MVLA 2.0 intervention, participants received 3 SMS text messaging or emails per week (Monday, Wednesday, and Thursday) inviting them to complete a specific activity. On Mondays, participants were invited to watch a relevant informational video for that week. On Wednesdays, participants were invited to visit the platform to read additional relevant educational information. On Thursdays, participants received a summary infographic of the week’s material. Figures S1 and S2 in Multimedia Appendix 1 provide examples of the infographics we used for week 3 (English and Spanish), and Table S1 in Multimedia Appendix 1 provides an outline of the intervention material and activities.

To deliver MVLA 2.0, we utilized the digital infrastructure of the Understanding America Study (UAS) platform. We created a participant-only website to provide access to weekly study content. The online platform for MVLA 2.0 included all study information, a discussion board, and downloadable infographics summarizing each week’s COVID-19 topic. Participants could select their preferred language (English or Spanish) to engage with the website and all study materials. Table S2 in Multimedia Appendix 1 shows a detailed description of the MVLA 2.0 intervention components (visual summaries, audio content, discussion board, and testimonial videos).

### Community Engagement, Recruitment, Timeline, and Other Intervention Logistics

We applied community-partnered research methods throughout the study. Recruitment involved community organization leaders and a group of parent ambassadors—parents who completed MVLA 1.0 and expressed interest in continuing to raise awareness by aiding with recruitment for MVLA 2.0. Recruitment was conducted through public school serving organizations located in East and South Los Angeles, communities with a high proportion of Hispanic families and disproportionately lower COVID-19 vaccination rates in children and youth based on Los Angeles County Department of Public Health Data [24]. We recruited participants using word-of-mouth and community partner social media sites to share the project flyer within their networks. The following organizations supported recruitment efforts by sharing our study flyer: Best Start LA, Coalition for Humane Immigrant Rights of Los Angeles, Communities in School, Families in School, Inner City Struggle, Great Public Schools Now, KIPP Public Charter Schools, Latino Equality Alliance, Mexican American Legal Defense and Education Fund, Mexican American Opportunity Foundation, Nuestra Voz (Our Voice), and Young Men's Christian Association of Metropolitan Los Angeles.

We planned to start recruitment when vaccines for children of ages 5 to 11 were newly released. Therefore, we tailored the intervention to address informational needs of Hispanic parents or caregivers of children in this age group. While the intervention information was relevant across all age groups, recruitment and intervention materials focused on households with children of ages 5 to 11 years as we anticipated the greatest impact among this age group. COVID-19 vaccines for children younger than 5 years were released in June 2022, and therefore, we updated intervention content to reflect up-to-date vaccine approval for the younger group (see Figures S1 and S2 in Multimedia Appendix 1).

We recruited parents and caregivers of Hispanic minors below 18 years of age who had access to a computer or cell phone to receive the intervention (inclusion criteria) via an online access open screener. Recruitment efforts were targeted not only to reach families with unvaccinated children of ages 5 to 11 years but also allowed interested families with at least one child below 18 years to participate in the intervention. While recruitment was focused among Los Angeles County parents, participants were not excluded if they resided in other neighboring areas or were from other racial or ethnic groups (no exclusion criteria).

We ended recruitment 2 weeks prior to starting the intervention to allow time to randomize all participants and enter their information for receiving SMS text messaging and emails into the digital platform. Figure S3 in Multimedia Appendix 1 shows the intervention implementation timeline. Participants first filled out an online screening survey to determine eligibility. Qualifying participants received an SMS text messaging and email inviting them to participate and directing them to the electronic consent. Upon consenting, participants were directed to the baseline survey. We did not establish specific eligibility criteria for sites or for individuals delivering the intervention, as the intervention was delivered digitally.

We used separate block randomization for cohorts, conducted by one UAS staff using Stata software, version 17 (StataCorp LLC). Randomized participants received MVLA 2.0 either at month 1 (intervention) or at month 2 (control). We used a 1:1 allocation ratio for randomization to the intervention and control groups. All participants were invited to complete a baseline and a 1-month follow-up survey. In the first month, the intervention group received educational material for 4 weeks, while the control group received a biweekly message reminding them of the number of days left until the beginning of month 2 when they were scheduled to start MiVacunaLA. After the intervention group completed the 4-week educational intervention, we sent all participants reminders twice a week for 2 weeks to complete the 1-month follow-up survey. The control group received the intervention after all groups completed the follow-up survey.

All intervention materials were delivered digitally. Participants completed the intervention activities in order. If a participant did not complete an intervention activity by the end of the week, they still received content for the following week. However, when participants attempted to access the next activity, they were prompted to first complete the missed activity. Participants had the flexibility to complete all intervention activities by the end of the month, with an additional 1-week grace period before the 1-month follow-up survey became available. Once the survey was available, participants could not complete additional intervention activities.

### Ethical Considerations

MiVacunaLA 2.0 was registered and approved by the University of California, Los Angeles Institutional Review Board (21‐000857). Participants completed an online informed consent form in their preferred language. Consent information was also available to participants as a PDF on the intervention platform for their reference. Participants’ contact information was collected in the screening survey. A study principal investigator worked with UAS staff to create identifiers so that all data downloaded from the UAS platform, and subsequently used in data analysis, were deidentified. Thus, no identification of individual participants in any images of the paper or supplementary material is possible. Only 1 principal investigator and 1 UAS staff member had access to participant identifiers, which were password-protected in an encrypted file, as well as access to pretrial randomization information. Study team members were blinded. After randomization, intervention and control participants started the intervention immediately (intervention arm) or in 2 months (waitlist control arm). Participants received a US $40 gift card sent electronically after completing all intervention activities.

Given this was a behavioral intervention and no concomitant care was provided during the intervention, there was minimal risk, and we did not collect data regarding harms. There were no significant changes to the trial after commencement or interim analysis during the data collection phase. We added information about US Food and Drug Administration–approved COVID-19 vaccines for children of ages 6 months to 4 years to the material during the design phase. During consent, participants were informed that they could decline participation at any time and could opt out of any intervention activity they did not feel comfortable completing. In addition to the detailed information provided on the curriculum and digital approach, those interested in accessing all the educational materials and survey documents to replicate this RCT may contact the corresponding author.

### Primary Outcomes

The primary outcome was self-reported vaccination status of household children in 3 age groups based on COVID-19 vaccine availability pre- and postintervention. The age groups used were (1) 6 months to 4 years, (2) 5 to 11 years, and (3) 12 to 17 years. The primary outcome was analyzed at the household level with at least 1 child in the specified age groups. The baseline survey asked for vaccination status for all household children. When participants confirmed a specific child’s vaccination, only the status of the household’s remaining nonvaccinated children was queried in the follow-up survey. We imputed the same vaccination status for the household expressed at baseline and the 1-month follow-up survey. For those with missing data, we assumed that if a household vaccinated 1 child in a specific age group, then the household’s children in that same age group were vaccinated at follow-up.

Table S3 in Multimedia Appendix 1 shows the vaccination status of qualifying households with children by age groups at baseline and follow-up for all participants, regardless of loss to follow-up. For those participants who did not complete the follow-up survey, we assumed that the vaccination status remained unchanged from baseline.

### Secondary Outcomes

Secondary outcomes included perceived change in COVID-19 vaccine knowledge and trust from baseline and follow-up. For vaccine knowledge, we asked participants to indicate whether the following statement was true or false: “After being fully vaccinated with the COVID-19 vaccine, your chances of being hospitalized or dying from COVID-19 are reduced at least by 90% if you contract the virus.” We coded the variable as equal to 1 if participants answered true, and 0 if they answered false or unsure. Second, we asked participants, “How would you rate your knowledge about the COVID-19 vaccine?” This variable was coded equal to 1 if participants answered adequate or superior knowledge, and equal to 0 if the answer was basic, minimal, or no knowledge.

We measured vaccine trust via 2 separate questions: “How much do you trust the governmental approval process to ensure the COVID-19 vaccine is safe for (1) the public and (2) children?” We coded this variable as equal to 1 for those who answered they trust government fully, mostly, or somewhat, and equal to 0 if they answered they do not trust the government for each question. We also measured participant engagement with the intervention regardless of the study arm. This analysis included all participants (intervention and waitlist control groups together) who interacted with the material on the digital platform and completed at least 1 activity. At the end of each week, we collected information on the perceived usefulness of the weekly video and in-platform information and participants’ familiarity with the information. We also tracked participants’ in-platform click-through rates of a link to the Los Angeles County Department of Public Health appointment portal for COVID-19 vaccination; whether participants downloaded infographics to share; and whether participants utilized audio files for written information and infographics sent midweek.

### Statistical Analysis

We evaluated efficacy using an intention-to-treat (ITT) approach. For the ITT analysis, we imputed the vaccination status for those who did not complete the follow-up survey with the same value entered at baseline. This conservative approach assumes no behavioral change among participants who were lost to follow-up. We used the same approach for the secondary outcomes.

We used a treatment-on-the-treated (TOT) approach as a sensitivity analysis. For the TOT analysis, we only included participants who completed the follow-up survey and whose vaccination status was reported at follow-up. We used a difference-in-difference (DID) model estimation for ITT and TOT approaches. Table S4 in Multimedia Appendix 1 shows the description of the DID model specification used for the statistical analysis.

To fit these models, we used a generalized linear model, using maximum likelihood estimation, with a binomial distribution and an identity link function to fit vaccination status as the dependent variable, with main fixed effects for both month (0=baseline and 1=follow-up) and intervention arm (0=control and 1=intervention), and an interaction term between month and intervention. The interaction term, defined specifically as Month×Intervention, represents the primary variable of interest and reflects the DID in the outcome variable. The models also accounted for repeated measures within the same household and utilized an unstructured correlation structure.

We ran Little’s Missing Completely at Random (MCAR) test to evaluate whether our data were missing completely at random. Subsequently, for the TOT analysis, we evaluated whether statistically significant differences exist between those who completed follow-up surveys and those who did not. We then modeled the TOT data using estimated weights based on significantly different characteristics between the 2 groups. This approach was used to help ensure we were not overestimating efficacy based on the characteristics of those who completed follow-up surveys. We estimated a Probit model where we regressed the attrition-related variable on those statistically significant variables between the 2 groups and created weights inverting these probabilities to estimate the intervention’s efficacy on COVID-19 vaccine uptake among household children within a specific age group. This RCT was reported using the CONSORT 2025 (Consolidated Standards of Reporting Trials) checklist (Checklist 1) [25].

## Results

### Demographic Characteristics for Overall, Control, and Intervention Samples

**Figure 1.**
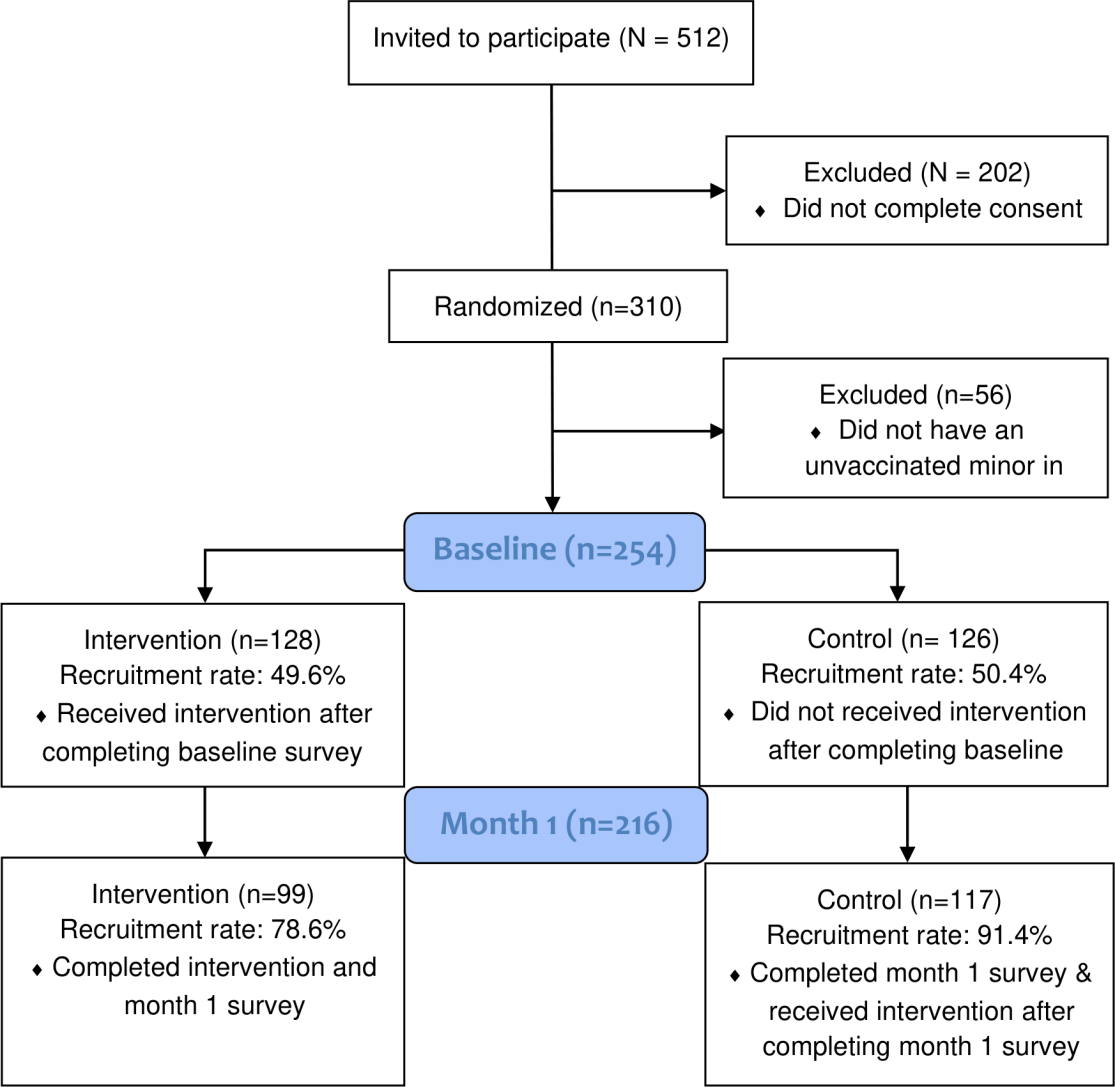
The CONSORT (Consolidated Standards of Reporting Trials) flow diagram for the intervention.

Figure 1 presents the CONSORT (Consolidated Standards of Reporting Trials) flow diagram for the intervention. Based on screening survey completion and study criteria, we invited 512 qualifying participants. Among those, 310 (60.5%) people consented to participate, completed the baseline survey, and were randomized to intervention or control groups. From those who consented and completed the initial baseline survey, we excluded 56 participants who reported no household minors in their baseline responses, which conflicted with their screener responses. Of the remaining 254 parents and caregivers in the baseline sample (126 and 128 participants in intervention and control groups, respectively), we had 216 participants who completed the 1-month follow-up survey (99 and 117 participants in intervention and control groups, respectively) with a retention rate of 85%. We had 17% missing data for the primary outcome or vaccine uptake. Of those, we imputed 37 for a total analytical sample of 247. We had 4% missing data for secondary outcomes or vaccine knowledge and trust. Our total analytic sample for secondary outcomes was 243.

Table 1 provides baseline demographic characteristics for the full sample and for control and intervention groups separately. The sample includes 254 participants with a household minor recruited at baseline. Most participants were Hispanic (n=230, 90.5%) and completed the intervention in English (n=207, 81.5%). Most participants had some college education or more (n=196, 77.2%), were employed (n=202, 79.5%), and over half (n=150, 59.1%) had an annual household income greater than US $50,000. Just over half had children aged 5 to 11 years (n=158, 62.2%), and about one-third had children aged 12 to 17 years (n=92, 36.2%). The average age of parents or caregivers was 33.8 (SD 6.3) years, and the average number of children in the household was 1.7 (SD 0.8). We evaluated whether differences in participant characteristics between study arms were statistically significant using a *t* test.

**Table 1.** Baseline demographic characteristics by overall, control, and intervention samples.

Characteristic	Overall (N=254)^a^	Control (n=128)	Intervention (n=126)	*P* value^b^
Age of parent, mean (SD)^c^	33.8 (6.3)	33.7 (6.2)	33.9 (6.4)	.68
Number of minors in household, mean (SD)	1.7 (0.8)	1.7 (0.9)	1.6 (0.7)	.82
Language, n (%)				.45
English	207 (81.5)	102 (79.7)	105 (83.3)	
Spanish	47 (18.5)	26 (20.3)	21 (16.7)	
Parent COVID-19 vaccination status, n (%)				.31
Vaccinated	95 (37.4)	51 (39.8)	44 (34.9)	
Not vaccinated	155 (61)	76 (59.4)	79 (62.7)	
Unsure/missing	4 (1.6)	1 (0.8)	3 (2.4)	
Ethnicity, n (%)				.89
Not Hispanic/Spanish origin or missing	24 (9.4)	11 (8.6)	13 (10.3)	
Mexican/Mexican American/Chicano	152 (59.8)	79 (61.7)	73 (57.9)	
Other Hispanic/Spanish origin^d^	78 (30.7)	38 (29.7)	40 (31.8)	
Born in the United States, n (%)				.18
Yes	187 (73.6)	99 (77.3)	88 (69.8)	
No	52 (20.5)	25 (19.5)	27 (21.4)	
Prefer not to respond/did not respond	15 (5.9)	4 (3.1)	11 (8.7)	
Highest education attained, n (%)				.99
Some high school or less	22 (8.7)	11 (8.6)	11 (8.7)	
High school graduate/GED^e^	30 (11.8)	15 (11.7)	15 (11.9)	
Some college or more	196 (77.2)	100 (78.1)	96 (76.2)	
Missing	6 (2.4)	2 (1.6)	4 (3.2)	
Employment status, n (%)				.10
Employed	202 (79.5)	107 (83.6)	95 (75.4)	
Unemployed	8 (3.2)	3 (2.3)	5 (4)	
Other^f^	44 (17.3)	18 (14.1)	26 (20.6)	
Household income, n (%)				.64
<$25,000	51 (20.1)	23 (18)	28 (22.2)	
$25,000-$49,000	46 (18.1)	25 (19.5)	21 (16.7)	
>$50,000	150 (59.1)	77 (60.2)	73 (57.9)	
Missing	7 (2.8)	3 (2.3)	4 (3.2)	
Health insurance status, n (%)				.24
Insured^g^	227 (89.4)	115 (89.8)	112 (88.9)	
Not insured/don’t know	27 (10.6)	13 (10.2)	14 (11.1)	
Marital status, n (%)				.40
Currently married	203 (79.9)	105 (82)	98 (77.8)	
Widowed/divorced/separated	20 (7.9)	9 (7)	11 (8.7)	
Never married	20 (7.9)	8 (6.3)	12 (9.5)	
Other^h^	11 (4.3)	6 (4.7)	5 (4)	
Type of household, n (%)				.61
Married with children	207 (81.5)	107 (83.6)	100 (79.4)	
Single/married without children	7 (2.8)	2 (1.6)	5 (4)	
Single with children	16 (6.3)	9 (7)	7 (5.6)	
Other^i^	24 (9.4)	10 (7.8)	14 (11.1)	
Any minors in household under 6 months of age, n (%)				
Yes^j^	8 (3.1)	5 (3.9)	3 (2.4)	.72
Any minors in household from 6 months to 4 years, n (%)				
Yes^j^	105 (41.3)	51 (39.8)	54 (42.9)	.63
Any minors in household from 5 to 11 years, n (%)				
Yes^j^	158 (62.2)	87 (68)	71 (56.4)	.06
Any minors in household from 12 to 17 years, n (%)				
Yes^j^	92 (36.2)	44 (34.4)	48 (38.1)	.54

a Excludes n=56 who reported no minors in their household at baseline.

b *P* value from χ2 tests (or Fisher exact tests, when appropriate) for categorical variables and Wilcoxon tests for continuous variables.

c Control group: n=125 parents had nonmissing age; intervention group: n=117 had nonmissing age.

d Includes Puerto Rican, Cuban, multiple ethnicities, and “other”.

e GED: general education development.

f Includes housekeeper, retired, disabled, temporary employment, student, and “other”.

g Includes government insurance, insurance through the Veteran Affairs, private insurance, and Medicare.

h Includes missing and cohabitation (common law marriage).

i Includes don’t know, prefer not to respond, missing, and “other”.

j All other participants indicated “no”.

There were no statistically significant differences between intervention and control groups in demographic characteristics. Table S3 in Multimedia Appendix 1 shows more intervention versus control households indicated at least 1 minor aged 5 to 11 years in their household (22.5% vs 19.5%) was vaccinated at baseline, while fewer intervention versus control households reported having at least 1 minor vaccinated at baseline in other age groups (5.6% vs 13.7% for minors 6 mo to 4 y; 27.1% vs 34.1% for minors 12 to 17 years old).

Table S5 in Multimedia Appendix 1 shows demographic characteristics at baseline for those completing the follow-up survey (216/254, 85%) and those who did not (38/254, 15%). Most significant demographic characteristics were associated with follow-up survey completion. Those who completed the follow-up survey were more likely to be unvaccinated, Hispanic, employed, US born, currently married or cohabitating, younger, with a smaller number of children at baseline. They were also more likely to have higher educational attainment and a household income greater than US $50,000. Table S5 also discusses weights considered for TOT estimation related to significant differences between follow-up completers and noncompleters.

### DID Model With the ITT Approach

Estimates from the DID model using the ITT approach for the primary outcomes related to vaccination are shown in Table 2 for households with children in each age group individually and all together. Table 2 shows vaccination status estimates for control and intervention groups at baseline and at follow-up in the regression analysis setting, alongside estimates of the difference within and between groups at pre- and postintervention. The control and intervention groups had overall baseline vaccination rates of 25.7% and 15.7%, respectively. At follow-up, the overall vaccination rates were 44.2% and 48.4%, respectively, among these groups. The estimate of interest in Table 2 is the DID estimate shown as “difference.” We found a statistically significant difference between intervention and control groups of 13.3% points (95% CI 0.3%-26.4%; *P*=.04) among children aged 5 to 11 years in change from pre- to postintervention. We did not find a significant DID for other age groups. Given ITT as the conservative estimate of intervention efficacy, we concluded that the intervention had a positive effect on COVID-19 vaccine uptake among the primary target group for this intervention, children aged 5 to 11 years.

**Table 2.** Difference-in-difference (DID) regression estimates for primary outcomes with the intention-to-treat (ITT) approach: vaccination status (N=247)^a^.

	Baseline, %	1-month follow-up, %	Change Δ (95% CI)	*P* value
6 months-4 years^b^				
Control	13.8	31.4	17.6 (7.0 to 28.2)	.001
Intervention	5.5	27.8	22.3 (11.1 to 33.5)	<.001
Difference	N/A^c^	N/A	4.7 (–10.7 to 20.1)	.55
5‐11 years^b^				
Control	19.5	34.5	14.9 (7.5 to 22.4)	<.001
Intervention	22.4	50.7	28.3 (17.6 to 38.9)	<.001
Difference	N/A	N/A	13.3 (0.3 to 26.4)	.04
12‐17 years^b^				
Control	34.1	50	15.9 (5.1 to 26.7)	.004
Intervention	27.1	47.9	20.8 (9.3 to 32.3)	<.001
Difference	N/A	N/A	4.9 (–10.9 to 20.7)	.54
All ages^b^				
Control	26.6	43.6	16.9 (10.3 to 23.6)	<.001
Intervention	21.9	46.3	24.4 (16.8 to 32.1)	<.001
Difference	N/A	N/A	7.5 (–2.6 to 17.7)	.15

a Simple (unadjusted) DIDs of vaccination of minors in household. Rates of vaccination are among those participants who indicated having at least 1 minor in the household within that age range and assume that anyone missing a 1-month follow-up had minors who remained unvaccinated. For those who reported a minor was vaccinated at baseline (and thus were not asked the vaccination question at 1 mo), we imputed the “yes” value to the 1-month follow-up, given that the question is not asked again at follow-up for this group. Models are estimated using a generalized linear model and maximum likelihood estimates.

b Sample sizes for each age group are as follows: n=105 households for 6 months-4 years; n=158 households for 5-11 years; n=92 households for 12‐17 years.

c N/A: not applicable.

Estimates of the DID model with the ITT approach for secondary outcomes related to vaccine knowledge and trust are shown in Table 3. We found a statistically significant difference between the intervention and control groups of 14.3% points (95% CI 5.0%‐23.7%; *P*=.003) preintervention to postintervention for trust in the governmental approval process to ensure the COVID-19 vaccine is safe for children. We did not find significant effects of the intervention on other secondary outcomes.

**Table 3.** Difference-in-difference (DID) regression estimates for secondary outcomes with the intention-to-treat (ITT) approach: vaccine knowledge and trust (N=243)^a^.

	Baseline, %	1-month follow-up, %	Change Δ (95% CI)	*P* value
...chances of being hospitalized or dying from COVID-19...^b^				
Control	52.9	65.1	12.2 (3.6 to 20.8)	.005
Intervention	50	65.1	15.0 (5.9 to 24.1)	.001
Difference	N/A^c^	N/A	2.8 (−9.8 to 15.4)	.66
...rate knowledge about COVID-19 vaccine...^d^				
Control	25.2	43.1	17.9 (8.9 to 26.9)	.001
Intervention	30.8	56.7	25.8 (15.9 to 35.8)	<.001
Difference	N/A	N/A	8.0 (–5.5 to 21.4)	.25
...trust governmental approval process... for public...^e^				
Control	81.3	89.4	8.1 (1.9 to 14.3)	.01
Intervention	74.2	90	15.8 (9.3 to 22.4)	<.001
Difference	N/A	N/A	7.7 (–1.3 to 16.7)	.09
...trust governmental approval process... for children...^e^				
Control	78.9	85.4	6.5 (0.7 to 12.4)	.03
Intervention	66.7	87.5	20.8 (13.6 to 28.1)	<.001
Difference	N/A	N/A	14.3 (5.0 to 23.7)	.003

a Simple (unadjusted) DID of knowledge and trust. Rates are among those participants who indicated having at least 1 minor in the household and assume that anyone missing a 1-month follow-up did not change their trust and knowledge from baseline. Models are estimated using a generalized linear model and maximum likelihood estimates.

b Variable coded equal to 1 if true, equal to 0 if false/unsure.

c N/A: not applicable.

d Variable coded equal to 1 if adequate/superior knowledge, equal to 0 if no/minimal/basic knowledge.

e Variable coded equal to 1 if fully/mostly/somewhat trust, equal to zero if do not trust.

### Participant Engagement

Table S6 in Multimedia Appendix 1 shows most participants (228/270, 84% to 228/257, 89%) found the video (provided on Monday) and educational information (provided on Wednesday) very/extremely useful. Materials in weeks 1 and 4 had the highest percentages of participants (236/266 and 220/247, 89%) expressing material was very or extremely useful. When asked about familiarity with that week’s provided information, only about half of the participants (108/266, 41% to 132/247, 53%) responded they were very/extremely familiar, with week 1 information showing the lowest percentage of participants (108/266, 41%) responding being very/extremely familiar with information.

Table S7 in Multimedia Appendix 1 summarizes participant engagement data. More than half of the participants (124/265, 47% to 147/252, 58%) clicked the specific link for making a COVID-19 vaccination appointment each week. Among those who completed that week’s activity, 89% (219/246) to 90% (227/252) of participants clicked to download the summary infographic to save/share. A large number of participants listened to the audio files from the written information sent on Wednesdays (681/1037, 66%) and the summary infographic sent on Thursdays (562/1037, 54%). Please note that percentages presented in Tables S6 and S7 are based on all participant information collected in our platform, regardless of whether the participant was included in our main analysis of primary and secondary outcomes.

### Sensitivity Analysis

A TOT sensitivity analysis with DID model estimation was used. Table S8 in Multimedia Appendix 1 presents estimates from the DID model for the primary vaccination outcome, both for individual age groups and collectively. The analysis revealed an 18.6 percentage point difference (95% CI) in vaccination uptake for children aged 5 to 11 years in the intervention group compared to controls (*P*=.02), and a 12.1 percentage point increase when considering all age groups (*P*=.04). Little’s MCAR test found evidence that data were not MCAR (*P*<.01). Weighted estimates from the DID model, which account for statistically significant differences between participants who completed the follow-up survey and those who did not, are provided in Table S9 in Multimedia Appendix 1. These weighted TOT estimates for children aged 5 to 11 years and the overall sample closely align with the unweighted estimates, showing increases of 20.1 percentage points (*P*=.01) and 12.2 percentage points (*P*=.04), respectively.

Similarly, TOT analyses of secondary outcomes related to vaccine knowledge and trust (Tables S10 and S11 in Multimedia Appendix 1) are consistent with the ITT results. Specifically, we found a larger increase in trust in the governmental approval process for children post intervention, with TOT unweighted and weighted estimates showing differences of 18.8 and 20.1 percentage points (*P*=.01), respectively. In addition, these tables indicate a significant difference of 10.8 and 10.6 percentage points (*P*=.05) in government approval processes for the public for unweighted and weighted TOT analyses, respectively. The detailed estimates using the TOT approach with the DID model are presented in Tables S8 to S11 in Multimedia Appendix 1.

## Discussion

### The Role of Community-Based Digital Interventions in Addressing Vaccine Hesitancy

In this study, we examined whether a community-based digital intervention could increase COVID-19 vaccination rates and trust in the governmental approval process to ensure vaccine safety in children aged 5 to 11 years. MVLA 2.0 significantly increased COVID-19 vaccine uptake in children aged 5 to 11 years and trust in the COVID-19 vaccine governmental approval processes for children. The MVLA 2.0 digital intervention demonstrated an effect on vaccine uptake comparable to the magnitude of effect observed in another such COVID-19-specific digital intervention [26], which found an adjusted absolute difference of 11.9 (95% CI 4.5‐19.3) percentage points in their 1-week intervention conducted during emergency room visits. Electronic health record approaches typically used in broader immunization efforts have demonstrated effect sizes between 4.2% (adjusted) and 7% (*P*=.05) [27,28]. These findings offer new insights into the role of community-informed and culturally tailored digital strategies, such as MVLA 2.0, in increasing trust in vaccines and improving vaccination rates. Furthermore, this digital intervention demonstrated the potential for scaling across diverse communities to address other pediatric vaccination gaps.

MVLA 2.0 intervention impact appears to reflect a combination of delivery, cultural tailoring, and user-centered design. Although we cannot rule out selection or reporting bias as contributors to the observed effects, interviews from MVLA 1.0 participants suggest that several components were likely impactful [23]. These included digitally accessible SMS text messaging delivering a web-based curriculum, with written educational content and videos reinforcing weekly material content over time. We also incorporated culturally tailored, real-time feedback to address parental concerns about COVID-19 vaccinations in children of ages 5 to 11 years and counter misinformation, disinformation, and myths prevalent in the Hispanic community. Finally, we applied user-centered design features such as a discussion board and voice-overs for written content to enhance engagement, accessibility, and adherence.

Although some progress for COVID-19 vaccination was made during the pandemic within the Hispanic community, trends in overall vaccinations remain low. Furthermore, national estimates on intent to get a COVID-19 vaccine among Hispanic groups have dropped from 18.6% to 11.4% between September 2024 and January 2025 [29]. Perhaps more concerning is the postpandemic decline in uptake of other childhood vaccines [2]. In California, where this intervention was conducted, statewide estimates on unvaccinated Hispanics have increased nearly 5% in the 4-month period between the end of September 2024 (15.7%) and the end of January 2025 (19.2%) alone [29]. A January 2025 article received media coverage over its analysis of 2019‐2022 vaccination data showing an overall decline in MMR and diphtheria, tetanus, and acellular pertussis vaccination rates across 11 states and, more worrisome, that three of those states had MMR statewide rates below herd immunity and broadly vulnerable to an outbreak [30]. Just weeks after these data were published, also widely reported, Texas experienced its largest measles outbreak in 3 decades, with the first US mortality from measles in a decade and the first pediatric mortality in 2 decades [31].

In addition, there are concerns that many of the impediments to COVID vaccination are similarly involved with the more recent downward trends in vaccination elsewhere, and some initial evidence to suggest these concerns may be warranted [32]. Because the MVLA 2.0 intervention was designed to be digital and culturally tailored, this model may have the potential for scaling across diverse communities to address other pediatric vaccination gaps. Future research is needed to explore the use of digital culturally tailored interventions such as MVLA 2.0 more broadly, as a cost-effective strategy to improve vaccination rates and reduce disparities in undervaccination for children.

Both intervention and control groups showed an increase in knowledge postintervention. Although the magnitude of this increase was not significantly different between groups for most outcomes, this study showed a significant intervention effect for increasing trust regarding the governmental approval process of the COVID-19 vaccine for children. A recent systematic review reported that health concerns, vaccine attributes, and mistrust were primary drivers of COVID-19 vaccine hesitancy, whereas trust and confidence, community and social factors, and demographics and identity were key factors associated with vaccine uptake [33]. Altogether, these broader trends in vaccine hesitancy and the associated causes reinforce the need for additional research into broad-based, easily disseminated multifactorial approaches to improve vaccine confidence.

Approaches, such as the mobile phone–centric format used in MVLA 2.0, may help promote vaccine acceptance, especially given the widespread cellphone and smartphone ownership. According to the Pew Research Center, 98% of Americans own some kind of cellphone and 91% some kind of smartphone. The penetration of ownership/type of usage is relatively high regardless of income (95% and 84%, respectively, those making under $30,000 per year) or ethnicity (97%/87% Black and 99%/93% Hispanic) and even age (94%/79% for those above 65 years of age) [34]. Furthermore, considerable support can be found in the literature for adopting digital, mobile-based interventions to change health behaviors, including immunization among adolescents [35], influenza vaccination among pediatric Hispanic patients in low-income urban areas [36], increasing human papillomavirus vaccine uptake among underserved high-risk populations [37], as well as culturally specific vaccine education to combat misinformation and improve vaccination behaviors [36,38-46]. Prior studies have recognized the ease of implementation as a benefit of mobile phone–delivered text-based interventions [36,38-53].

SMS text messaging interventions were successful in increasing influenza vaccination rates among Hispanic patients in low-income urban areas [36] and in increasing human papillomavirus vaccine uptake among underserved high-risk populations [37], as well as culturally specific vaccine education to combat misinformation [36,38-46]. Prior studies have recognized the ease of implementation as a benefit of mobile phone–delivered text-based interventions [36,38-53].

### Study Limitations and Suggested Future Work

This study has several limitations. We used self-reported vaccination status, which is subject to over- or under-reporting. Parents may have over-reported vaccination due to social desirability bias, particularly after participating in a provaccine educational intervention. The lack of objective verification through medical records or vaccination databases reduces confidence in the primary findings. However, the target population for this intervention may be less likely to provide official records due to concerns with immigration and documentation status. Thus, building trust to ensure the collection of official vaccination records for future digital vaccine acceptance interventions is key.

Lower baseline vaccination rates in the intervention group may overestimate efficacy. Due to a 15% attrition rate and systematic differences between completers and noncompleters, the ITT analysis may be compromised, despite attempts to adjust for these differences using weighting in the TOT analysis. The assumption that vaccinating 1 child in an age group translates to all children in that household being vaccinated for those with missing data could lead to inflated effect sizes. In this study, individual children in a household did not have unique IDs; therefore, we were unable to link responses between baseline and follow-up. Thus, future work should aim to analyze vaccination uptake at the individual level. Furthermore, CIs in our results were wide in relation to our primary (vaccination status of children aged 5-11 years) and secondary outcomes (trust in the government process for the approval of COVID-19 vaccines for children). This variability may suggest that estimates of intervention effect lack precision, likely explained by the small sample size. Thus, future studies warrant a larger sample size to provide a more precise estimate of the efficacy of a digital intervention on vaccine uptake and trust.

Although a strength of this study included using a community-based approach to recruit populations typically “under-represented” in research, this also may limit the generalizability of our findings. First, we primarily recruited participants who had already engaged with community organizations and were willing to participate in vaccine research, which could create selection bias. This population could differ systematically from the broader Hispanic community in terms of health engagement, technology access, and baseline vaccine attitudes. Second, most participants completed the intervention in English, so results may not generalize to households who prefer to communicate in Spanish. Interestingly, during the MVLA 1.0 intervention, 79% of participants completed the intervention in Spanish [22]. This shift in language predominance might be explained by differences in the community partner organizations engaged in MVLA 2.0 and the populations they serve. Third, there may have been a bias in participants willing to be screened for eligibility to participate. Future digital intervention studies should work closely with community organizations to incorporate a screening method where the community organization can confirm whether participants are affiliated with their organization to improve recruitment tracking.

Future directions for vaccine interventions should emphasize the development of culturally tailored community-based digital strategies to build trust in vaccines. This includes actively engaging with community leaders and advocates to ensure that interventions are relevant and resonate with the target population. Addressing misinformation could involve using mobile platforms to provide accurate, accessible information and counteract false narratives in the same modality in which they are being disseminated. In addition, improving vaccination behaviors might be achieved by integrating behavioral insights to identify and overcome specific barriers to vaccination within the community. Scaling these strategies to diverse populations is critical to address the challenge of decreasing vaccination rates.

### Conclusions

The findings from this RCT evaluating MVLA 2.0 (a culturally tailored, community-based, mobile phone–delivered intervention) demonstrate higher COVID-19 vaccine uptake among Hispanic children and increased parental trust in the governmental vaccine approval process compared with the control group. This study is innovative in combining community-informed content, real-time culturally tailored feedback, and a fully automated, mobile phone–centric delivery model. It adds to existing literature by testing a culturally tailored digital curriculum in a community setting and showing improvements in both uptake and trust. These findings provide rigorous evidence that scalable, low-cost digital strategies can address vaccine confidence and uptake in populations with persistent gaps in vaccine acceptance. Real-world implications include the portability and adaptability of this approach across diverse communities and settings—urban or rural—and its feasibility and potential for broader applicability and scalability to support public health vaccination campaigns.

## Supplementary material

10.2196/78103 Multimedia Appendix 1 Examples of the culturally tailored infographics used in the MiVacunaLA 2.0 digital intervention.

10.2196/78103 Checklist 1 CONSORT checklist.

## Abbreviations

CONSORT: Consolidated Standards of Reporting Trials
DID: difference-in-difference
HPV: human papillomavirus
ITT: intention-to-treat
MCAR: Missing Completely at Random
MMR: measles-mumps-rubella
MVLA: MiVacunaLA/MyShotLA
RCT: randomized controlled trial
TOT: treatment-on-the-treated
UAS: Understanding America Survey
UCLA: University of California, Los Angeles
