# Supplementary material for: Digital Intervention (MiVacunaLA 2.0) to Promote COVID-19 Vaccine Acceptance Among Hispanic Children: Community-Based Randomized Controlled Trial
Source: J Med Internet Res. 2026 Mar 30;28:e78103. doi: 10.2196/78103 (PMC13035084; doi:10.2196/78103)
Supplement: Multimedia Appendix 1 [file jmir-v28-e78103-s001.pdf]

Multimedia Appendix

Figure A1 – Week 3 infographic (English)

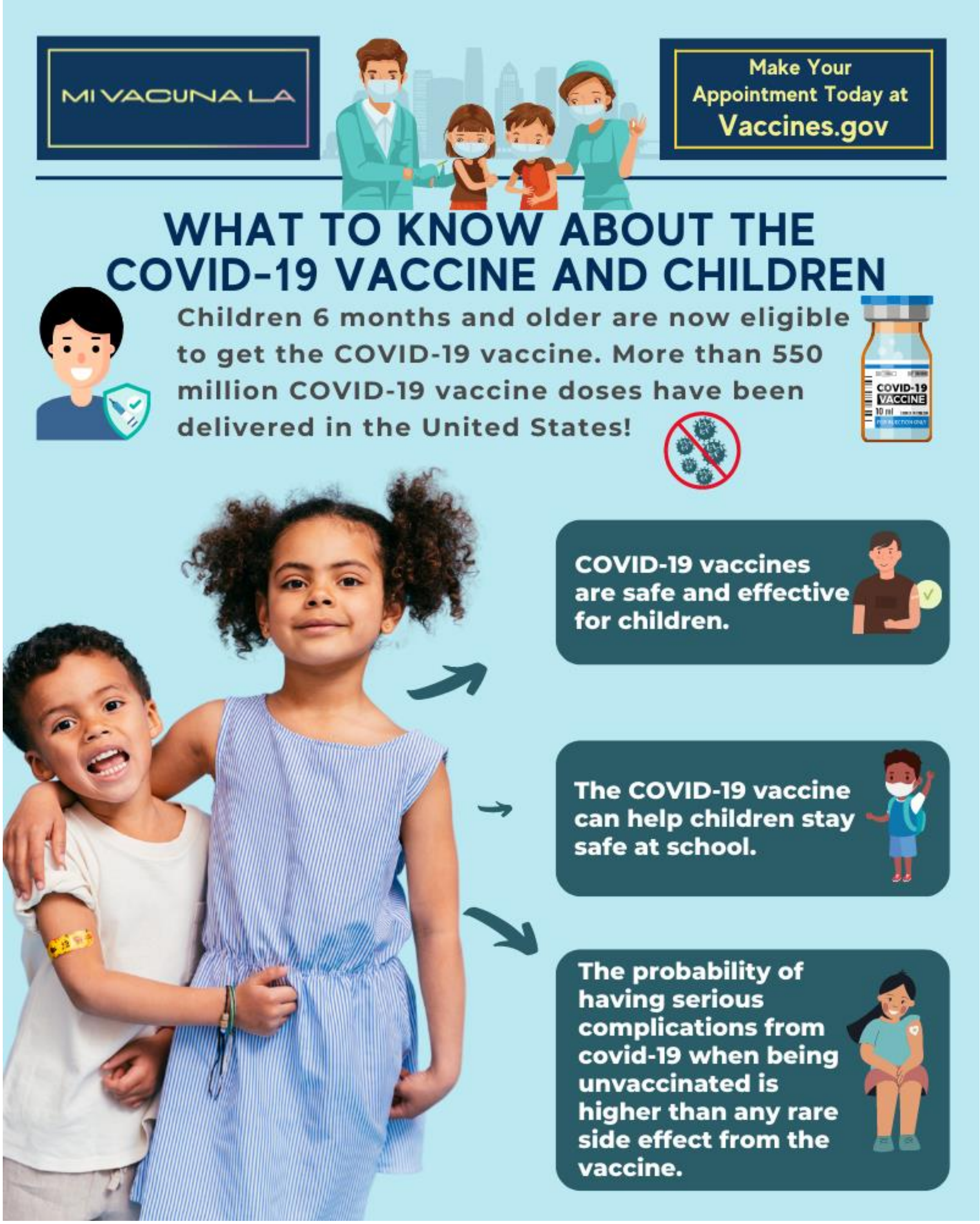

Figure A2 – Week 3 infographic (Spanish)

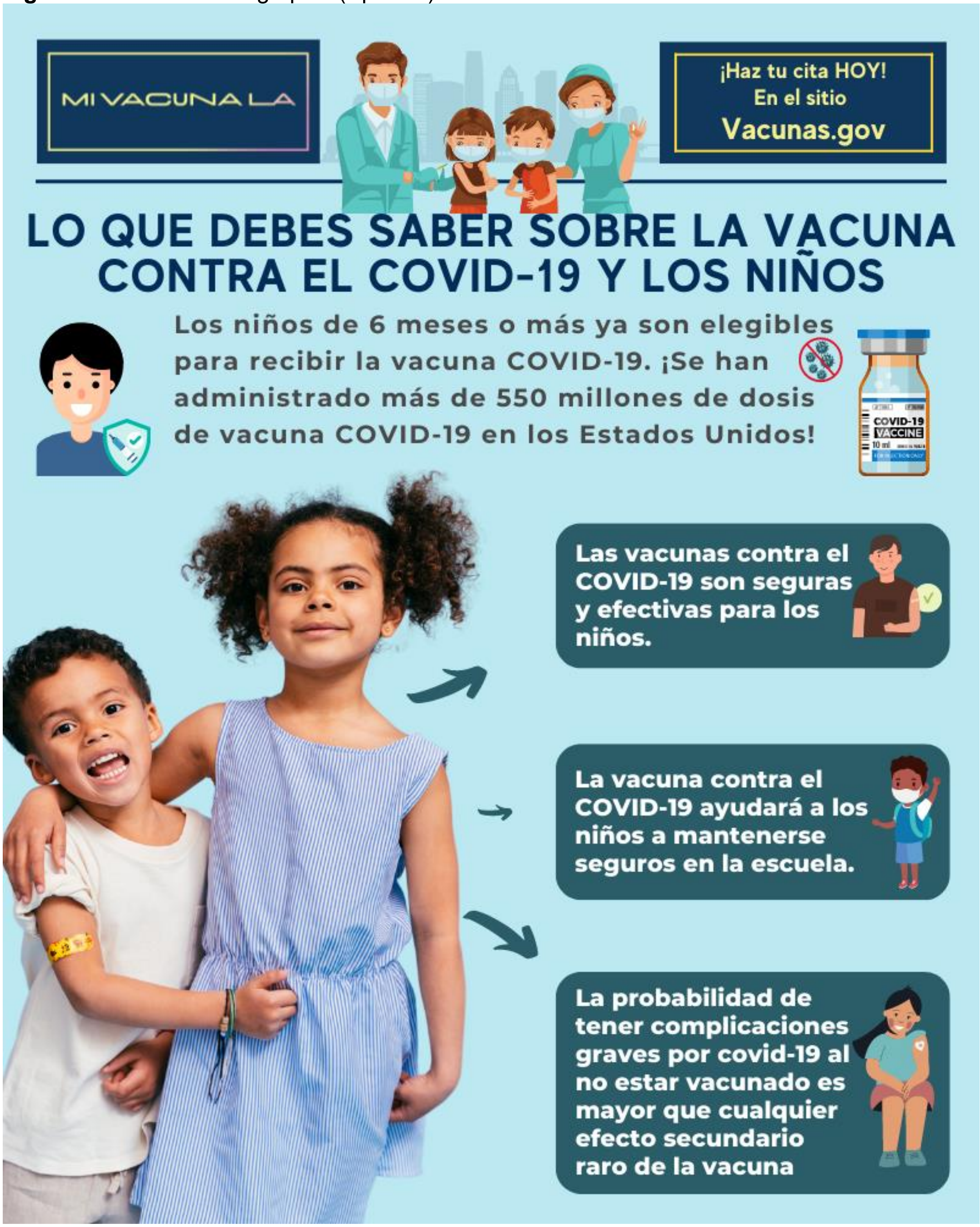

Table A1. Outline of program material and activities

Week	Activities[a]
Week 1	TOPIC: Vaccine Function and Health Experts<br>• Video 1 Title: Program introduction – Drs. BLINDED FOR REVIEW (New video)<br>• Information Topic: What is COVID-19 and why should we get the COVID-19 vaccine<br>• Related Infographic
Week 2	TOPIC: Vaccine and Myths<br>• Video 2 Title: Myths and truths – Dr. BLINDED FOR REVIEW (same as MiVacunaLA 1.0)<br>• Information Topic: What to Know about COVID-19 Vaccine Myths<br>• Related Infographic
Week 3	TOPIC: Vaccine and Children<br>• Video 3 Title: Vaccines and Children – Dr. BLINDED FOR REVIEW (New video)<br>• Information Topic: What to know about the COVID-19 vaccine and children<br>• Related Infographic
Week 4	TOPIC: , How to get the COVID Vaccine<br>• Video 4 Title: Parent Testimonial – Elsy G.<br>• Information Topic: 5 Things to Know to Get the COVID-19 Vaccine in Los Angeles County (new video)<br>• Related Infographic

[a]Message to invite participants to review video went out on Mondays, to review written information (with audio file) went out on Wednesdays, and to review infographic (with audio file) on Thursdays. Participants received also a reminder on Sundays to complete all activities of the week.

Table A2. MiVacunaLA 2.0 intervention components

Component	Description
Visual Summaries	We worked with a professional communications company to design infographics based on weekly content developed by the PIs. See an example of our infographics in the online Appendix (Figures A1 and A2, English and Spanish respectively). Participants received an email and a text message each week linking to the infographic visual summarizing of that week's important highlights and takeaways. Infographics also featured hyperlinks and QR codes to local school and public health vaccination sites for COVID-19 vaccine information and scheduling. We held a community meeting and invited prior participants who expressed interest in being contacted for future studies to review and revise infographics and the intervention's educational content specific to COVID-19 vaccination in children. We also asked community participants to provide feedback on Spanish language use, the summarizing of weekly material on infographics, and content needed to improve vaccine confidence in parents.
Audio Content	We provided voiceovers in English and Spanish of written content to address literacy needs and enhance accessibility. Recorded audios aided in facilitating participants' ability to listen to weekly written material.
Discussion Board	At the end of the intervention, we created a digital discussion board for participants to submit questions related to the COVID-19 vaccine as well as our intervention. During Week Three, participants were notified and given discussion board access for submissions and given three days in which to submit questions. The discussion board was moderated by the study team, with all questions and answers reviewed before postings could be viewed by other participants. Questions were answered by a member of the study team and reviewed for accuracy by a study team physician. Answers were posted during the final week of the intervention with participants notified afterwards via text message that answers to submitted questions were available for viewing.
Testimonial Videos	During the fourth week of the intervention, participants received a link to a bilingual English and Spanish video testimonial from a Latina mother living in Los Angeles who vaccinated her child. In the testimonial, the mother describes why she decided to vaccinate her child, her experience vaccinating her child, and recommendations she had for other parents unsure of their decision to vaccinate.

Figure A3. Intervention timeline

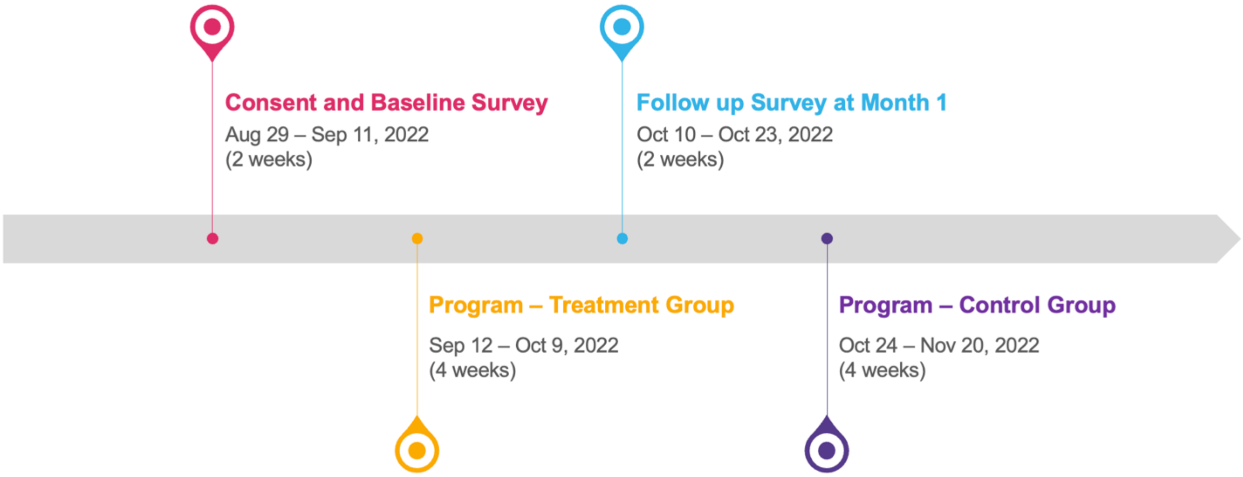

Table A3. Frequencies of vaccination at baseline and 1-month[a]

	Intervention		Control	
	Baseline	1 Month	Baseline	1 Month
**Minor 6 months-4 years, n (%)**	**N=54**		**N=51**	
Yes	3 (5.6)	15 (27.8)	7 (13.7)	16 (31.4)
No	49 (90.7)	39 (72.2)	43 (84.3)	35 (68.6)
Missing	2 (3.7)	0 (0.0)	1 (2.0)	0 (0.0)
**Minor 5-11 years, n (%)**	**N=71**		**N=87**	
Yes	16 (22.5)	36 (50.7)	17 (19.5)	30 (34.5)
No	53 (74.7)	35 (49.3)	70 (80.5)	57 (65.5)
Missing	2 (2.8)	0 (0.0)	0 (0.0)	0 (0.0)
**Minor 12-17 years, n (%)**	**N=48**		**N=44**	
Yes	13 (27.1)	23 (47.9)	15 (34.1)	22 (50.0)
No	35 (72.9)	25 (52.1)	29 (65.9)	22 (50.0)
Missing	0 (0.0)	0 (0.0)	0 (0.0)	0 (0.0)

[a] Here we include those participants who indicated that had at least one minor in their household within the given age-range. If participant reported the minor vaccinated at baseline (and was thus not asked the question at their 1-month follow-up), we “imputed” the vaccination status at the 1-month follow-up. For those households with an unvaccinated child that did not complete the follow up survey, we imputed a non-vaccination status for the child, so these are different age group samples that were considered for the Intention to Treatment (ITT) analysis.

Table A4. Difference-in-difference (did) model specification description

**Equation Specification**
$Y_{i,t} = \beta_0 + \beta_{treat} Treat + \beta_{post} Post + b_{int}(PostXTreat) + \propto_i + \varepsilon_{i,t}$
**Equation Description**
$Y_{i,t}$ represents the value of the dependent variable for the i_{th} participant in the t_{th} period. In the equation i = 1, 2,...,I_t and t = 1, 2. I_t denotes sample size of the t_{th} period.
β_0 is a vector of constants.
α_i captures individual unobserved characteristics (individual fixed effects).
ε_{it} represents the error terms for the i_{th} person in the t_{th} period.
Treat represents a variable that is equal to one if the person is in the intervention group, and equal to zero otherwise.
Post represents a variable that is equal to zero for Period 1 (baseline) and equal to one for Period 2 (postintervention, follow-up survey at month 1).
Post × *Treat* denotes the variable of interest that reflects the DID in the outcome variable Y.

Table A5. Baseline demographic characteristics by overall, completed follow-up survey, and non-completed follow-up survey samples[a]

Characteristic	Overall (N=254)	Non-Completed (N=38)	Completed (N=216)	p-value[b]
Age of parent (mean, SD)[c]	33.8 (6.3)	36.3 (6.9)	33.5 (6.1)	0.0143
No. Of minors in household (mean, SD)	1.7 (0.8)	1.9 (0.8)	1.6 (0.8)	0.0127
**Language, n (%)**				
English	207 (81.5)	28 (73.7)	179 (82.9)	0.1787
Spanish	47 (18.5)	10 (26.3)	37 (17.1)	
**Parent covid-19 vaccination status, n (%)**				
Vaccinated	95 (37.4)	19 (50.0)	76 (35.2)	0.0012
Not vaccinated	155 (61.0)	15 (39.5)	140 (64.8)	
Unsure	2 (0.8)	2 (5.3)	0 (0.0)	
Missing	2 (0.8)	2 (5.3)	0 (0.0)	
**Ethnicity, n (%)**				
Not Hispanic/Spanish origin	18 (7.1)	6 (15.8)	12 (5.6)	0.0180
Mexican/Mexican American/Chicano	152 (59.8)	15 (39.5)	137 (63.4)	
Other Hispanic/Spanish origin	78 (30.7)	11 (29.0)	67 (31.0)	
Missing	6 (2.4)	6 (15.8)	0 (0.0)	
**Born in the United States, n (%)**				
Yes	187 (73.6)	18 (47.4)	169 (78.2)	0.0030
No	52 (20.5)	10 (26.3)	42 (19.4)	
Prefer not to respond	9 (3.5)	4 (10.5)	5 (2.3)	
Missing	6 (2.4)	6 (15.8)	0 (0.0)	
**Highest education attained, n (%)**				
Some high school or less	22 (8.7)	9 (23.7)	13 (6.0)	0.0010
High school graduate/GED	30 (11.8)	2 (5.3)	28 (13.0)	
Some college or more	196 (77.2)	21 (55.3)	175 (81.0)	
Missing	6 (2.4)	6 (15.8)	0 (0.0)	
**Employment status, n (%)**				
Employed	202 (79.5)	19 (50.0)	183 (84.7)	0.0045
Unemployed	8 (3.2)	2 (5.3)	6 (2.8)	
Other[e]	33 (13.0)	9 (23.7)	24 (11.1)	
Don't know/prefer not to respond	5 (2.0)	2 (5.3)	3 (1.4)	
Missing	6 (2.4)	6 (15.8)	0 (0.0)	
**Household income, n (%)**				
< $25,000	51 (20.1)	15 (39.5)	36 (16.7)	0.0005
$25,000 - $49,000	46 (18.1)	6 (15.8)	40 (18.5)	
> $50,000	150 (59.1)	11 (29.0)	139 (64.4)	
Missing	7 (2.8)	6 (15.8)	1 (0.5)	
**Health insurance status, n (%)**				
Insured[f]	227 (89.4)	31 (81.6)	196 (90.7)	0.0532
Not insured	21 (8.3)	4 (10.5)	17 (7.9)	

Don't know/prefer not to respond	6 (2.4)	3 (7.9)	3 (1.4)	
**Marital status, n (%)**				
Currently married	203 (79.9)	18 (47.4)	185 (85.7)	0.0005
Cohabitation (common law marriage)	5 (2.0)	1 (2.6)	4 (1.8)	
Widowed/divorced/separated	20 (7.9)	5 (13.2)	15 (6.9)	
Never married	20 (7.9)	8 (21.0)	12 (5.6)	
Missing	6 (2.4)	6 (15.8)	0 (0.0)	
**Type of household, n (%)**				
Married with children	207(81.5)	21 (55.3)	186 (86.1)	0.0016
Single/married without children	7 (2.8)	0 (0.0)	7 (3.2)	
Single with children	16 (6.3)	3 (7.9)	13 (6.0)	
Other	4 (1.6)	2 (5.3)	2 (0.9)	
Don't know/prefer not to respond	13 (5.1)	6 (15.8)	7 (3.2)	
Missing	7 (2.8)	6 (15.8)	1 (0.5)	
**Any minors in household under 6 months, n (%)**				
Yes	8 (3.1)	2 (5.3)	6 (2.8)	0.3419
No	246 (96.9)	36 (94.7)	210 (97.2)	
**Any minors in household 6 months-4 years, n (%)**				
Yes	105 (41.3)	16 (42.1)	89 (41.2)	0.9171
No	149 (58.7)	22 (57.9)	127 (58.8)	
**Any minors in household 5-11 years, n (%)**				
Yes	158 (62.2)	22 (57.9)	136 (63.0)	0.5524
No	96 (37.8)	16 (42.1)	80 (37.0)	
**Any minors in household 12-17 years, n (%)**				
Yes	92 (36.2)	16 (42.1)	76 (35.2)	0.4131
No	162 (63.8)	22 (57.9)	140 (64.8)	

[a] Notes on TOT weights: we estimate our TOT using weights with most variables that show a statistically significant difference at least at the 95 percent confidence level. We do not include ethnicity in our weights estimation given our recruitment was focused on a specific ethnic group. We also do not include U.S. born as a weights estimation variable because when we collapse responses, the majority prefer not to respond or are missing, and thus no longer significantly different across those who completed and those who did not complete the follow-up survey at the 95 percent confidence level.
[b] p-value from chi-square tests (or Fisher's exact tests, when appropriate) for categorical variables and Wilcoxon tests for continuous variables
[c] Non-completers group: N=29 parents had non-missing age; Completed group: N=213 had non-missing age
[d] Includes: Puerto Rican, Cuban, multiple ethnicities, and "other"
[e] Includes: Housekeeper, Retired, Disabled, Temporary Employment, Student, and "Other"
[f] Includes: government insurance, insurance through the VA, private insurance, Medicare

Table A6. Usefulness and familiarity with program material by week[a]

	Week 1	Week 2	Week 3	Week 4	Total
**Usefulness of video**					
Very Useful or Extremely useful	228	228	216	220	892
Percent	(84.44)	(88.72)	(85.38)	(87.65)	(86.51)
Total Completed	270	257	253	251	1031
**Usefulness of information**					
Very Useful or Extremely useful	236	213	219	220	888
Percent	(88.72)	(83.86)	(86.91)	(89.06)	(87.15)
Total Completed	266	254	252	247	1019
**Familiarity with information covered**					
Very Familiar or Extremely Familiar	108	122	127	132	489
Percent	(40.60)	(48.03)	(50.40)	(53.44)	(47.99)
Total Completed	266	254	252	247	1019

[a] We include total of participants who answered, "very useful or extremely useful" (other categories: not at all useful, slightly useful, and moderately useful) and "very familiar of extremely familiar" (other categories: Not at all familiar, slightly familiar, moderately familiar), percentages of total of participants in those categories in parenthesis, and total of participants who completed the weekly activity.

Table A7. Total of clicks on make appointment, share infographic, information audio and infographic audio[a]

	Week 1	Week 2	Week 3	Week 4	Total
**Total Clicked Make Appointment**	124	136	147	137	544
Percent (%)	(46.79)	(53.54)	(58.33)	(55.69)	(53.49)
Total Completed	265	254	252	246	1,017
**Total Clicked to Share/Save Infographic**	237	228	227	219	911
Percent (%)	(89.43)	(89.76)	(90.08)	(89.02)	(89.58)
Total Completed	265	254	252	246	1,017
**Total Clicked Information Audio**	182	166	175	158	681
Percent (%)	(66.67)	(63.85)	(69.17)	(62.95)	(65.67)
Total Completed	273	260	253	251	1,037
**Total Clicked Infographic Audio**	150	142	147	123	562
Percent (%)	(54.95)	(54.62)	(58.10)	(49.00)	(54.19)
Total Completed	273	260	253	251	1,037

[a] We provide statistics by Week and Total (with percentages and total of participants completing that week activity). Total clicked denotes the total of participants who clicked, percentages of total completed are shown in parenthesis, and total completed denotes the total number of participants who completed the activity for that week.

Table A8. DID regression estimates for primary outcome with TOT approach: vaccination status[a]

	Baseline	1-month follow-up	Change Δ (95% CI)	P
**6 months-4 years[b], (%)**				
Control	13.0%	33.2%	20.1% (8.5%, 31.8%)	0.0007
Intervention	2.3%	32.3%	30.0% (15.7%, 44.2%)	<.0001
Difference			9.8% (-8.6%, 28.2%)	0.2949
**5-11 years[b], (%)**				
Control	17.5%	34.6%	17.1% (8.7%, 25.4%)	<.0001
Intervention	19.6%	55.4%	35.7% (23.2%, 48.3%)	<.0001
Difference			18.6% (3.6%, 33.7%)	0.0154
**12-17 years[b], (%)**				
Control	35.0%	54.0%	19% (6.8%, 31.3%)	0.0023
Intervention	22.2%	50.0%	27.8% (13.2%, 42.4%)	0.0002
Difference			8.7% (-10.4%, 27.8%)	0.3697
**All ages[b], (%)**				
Control	25.7%	44.5%	18.8% (11.6%, 26.1%)	<.0001
Intervention	17.5%	48.5%	30.9% (21.7%, 40.1%)	<.0001
Difference			12.1% (0.4%, 23.8%)	0.0426

[a] Simple (unadjusted) difference-in-differences of vaccination of minors in household. Rates of vaccination are among those participants who indicated having at least 1 minor in the household within that age range and includes only those participants with a 1-month follow-up. For those who reported a minor was vaccinated at baseline (and thus were not asked the vaccination questing at 1-month), we imputed the "yes" value to the 1-month follow-up. Model estimated using a generalized linear model and maximum likelihood estimates.

[b] Sample sizes for each age group are: N=89 households for 6 months-4 years; N=136 households for 5-11 years; N=76 households for 12-17 years, and N=210 households for all ages.

Table A9. DID regression estimates for primary outcome with TOT approach, weighted: vaccination status[a]

	Baseline	1-Month Follow-up	Change Δ (95% CI)	P
**6 months-4 years[b] (%)**				
Control	12.1%	30.3%	18.3% (7.3%, 29.3%)	0.0011
Intervention	2.4%	33.3%	30.9% (16.2%, 45.6%)	<.0001
Difference			12.6% (-5.7%, 30.9%)	0.1781
**5-11 years[b] (%)**				
Control	19.2%	35.0%	15.8% (7.9%, 23.7%)	<.0001
Intervention	20.9%	56.8%	35.9% (23.2%, 48.5%)	<.0001
Difference			20.1% (5.2%, 35.0%)	0.0083
**12-17 years[b] (%)**				
Control	38.2%	57.0%	18.8% (6.4%, 31.2%)	0.0029
Intervention	23.9%	48.8%	24.9% (10.6%, 39.2%)	0.0006
Difference			6.1% (-12.8%, 25.0%)	0.5264
**All ages[b] (%)**				
Control	28.2%	46.2%	18.0% (11.0%, 25.1%)	<.0001
Intervention	19.1%	49.4%	30.3% (21.0%, 39.5%)	<.0001
Difference			12.2% (0.6%, 23.9%)	0.0394

[a] Simple (unadjusted) difference-in-differences of vaccination of minors in household. Rates of vaccination are among those participants who indicated having at least 1 minor in the household within that age range. For those who reported a minor was vaccinated at baseline (and thus were not asked the vaccination questing at 1-month), we imputed the "yes" value to the 1-month follow-up given that question is not asked again at follow-up for this group. Model estimated using a generalized linear model and maximum likelihood estimates. Weights were calculated via a probit regression model with completed (yes/no) as the dependent variable, and parent vaccination status, education level, employment status, household income, marital status, age of parent, and number of minors in the household as independent variables.

[b] Sample sizes for each age group are: N=89 households for 6 months-4 years. N=136 households for 5-11 years; N=76 households for 12-17 years, and N=210 households for all ages.

Table A10. DID regression estimates for secondary outcomes with TOT approach: knowledge and trust on vaccines (N=210)[a]

	Baseline	1-Month Follow-up	Change Δ (95% CI)	P
**…chances of being hospitalized or dying from COVID-19…[b]**				
Control	54.9%	67.7%	12.9% (3.3%-22.4%	0.0082
Intervention	48.5%	67.0%	18.6% (7.4%-29.7%)	0.0012
Difference			5.7% (-9.0%-20.4%)	0.4475
**…rate knowledge about COVID-19 vaccine…[c]**				
Control	24.8%	45.0%	20.2% (10.1%-30.2%)	<.0001
Intervention	32.0%	63.9%	32.0% (20.0%-43.9%)	<.0001
Difference			11.8% (-3.9%-27.4%)	0.1397
**…trust governmental approval process… for public…[d]**				
Control	81.4%	90.2%	8.8% (1.9%-15.6%)	0.0121
Intervention	72.2%	91.8%	19.6% (11.7%-27.5%)	<.0001
Difference			10.8% (0.4%-21.3%)	0.0425
**…trust governmental approval process… for children…[d]**				
Control	77.9%	84.8%	7.0% (0.5%-13.4%)	0.0352
Intervention	62.9%	88.7%	25.8% (17.1%-34.5%)	<.0001
Difference			18.8% (8.0%-29.7%)	0.0007

[a] Simple (unadjusted) difference-in-differences of trust and knowledge. Rates are among those participants who indicated having at least 1 minor in the household and makes the assumption that anyone missing a 1-month follow-up did not change in their trust and knowledge from baseline. Model estimated using a generalized linear model and maximum likelihood estimates.
[b] Variable coded equal to 1 if true, equal to zero if false/unsure.
[c] Variable coded equal to 1 if adequate/superior knowledge, equal to zero if no/minimal/basic knowledge.
[d] Variable coded equal to 1 if fully/mostly/somewhat trust, equal to zero if do not trust.

Table A11. DID regression estimates for secondary outcomes with TOT approach, weighted: knowledge and trust on vaccines (N=210)[a]

	Baseline	1-Month Follow-up	Change Δ (95% CI)	P
**…chances of being hospitalized or dying from COVID-19…[b]**				
Control	54.7%	68.3%	13.6% (4.1%-23.0%)	0.0048
Intervention	48.0%	67.8%	19.7% (8.0%-31.5%)	0.0010
Difference			6.1% (-8.9%-21.2%)	0.4248
**…rate knowledge about COVID-19 vaccine…[c]**				
Control	24.3%	43.0%	18.7% (8.4%-29.2%)	0.0004
Intervention	32.0%	64.8%	32.9% (20.4%-45.3%)	<.0001
Difference			14.2% (-2.0%-30.3%)	0.0857
**…trust governmental approval process… for public…[d]**				
Control	82.2%	90.4%	8.2% (1.7%-14.7%)	0.0139
Intervention	73.5%	92.3%	18.8% (10.9%-26.7%)	<.0001
Difference			10.6% (0.4%-20.8%)	0.0414
**…trust governmental approval process… for children…[d]**				
Control	78.3%	84.8%	6.5% (0.3%-12.7%)	0.0396
Intervention	62.6%	89.2%	26.6% (17.6%-35.6%)	<.0001
Difference			20.1% (9.2%-31.0%)	0.0003

[a] Simple (unadjusted) difference-in-differences of trust and knowledge. Rates are among those participants who indicated having at least 1 minor in the household and makes the assumption that anyone missing a 1-month follow-up did not change in their trust and knowledge from baseline. Model estimated using a generalized linear model and maximum likelihood estimates. Weights were calculated via a probit regression model with completed (yes/no) as the dependent variable, and parent vaccination status, education level, employment status, household income, marital status, age of parent, and number of minors in the household as independent variables.

[b] Variable coded equal to 1 if true, equal to zero if false/unsure.

[c] Variable coded equal to 1 if adequate/superior knowledge, equal to zero if no/minimal/basic knowledge.

[d] Variable coded equal to 1 if fully/mostly/somewhat trust, equal to zero if do not trust.
